# Super large cardiac hemangioma in right atrium and inferior vena cava: case report

**DOI:** 10.1186/s13019-019-1016-6

**Published:** 2019-11-05

**Authors:** C. J. Perez Rivera, R. Figueroa-Casanova, C. E. Ochoa Bonet, A. González-Orozco

**Affiliations:** Department of Cardiovascular Surgery, Medical Doctor, Clinica Avidanti, Av. 19 #10 315, Ibagué, Tolima Colombia

**Keywords:** Cardiac hemangioma, Right ventricular prolapse, Jehovah’s witness, Case report

## Abstract

**Background:**

Cardiac hemangiomas are rare cardiac neoplasia usually diagnosed in autopsies, from being asymptomatic to debuting with sudden death. The largest hemangioma published in the literature is of 130 mm size, we present the following case of a successful cardiac hemangioma excision of 280 × 35 mm in size, diagnosed due to recurrent cardiac symptoms.

**Case presentation:**

A 48-year-old female patient, Jehovah’s Witness, with no previous diagnoses, is admitted due to recurrent syncopal episodes in the previous months. A transthoracic echocardiogram diagnosed a tumor in the right atrium and inferior vena cava producing a diastolic right ventricular, with preservation of the left ventricular ejection fraction at 55%. Given the high mortality risk, a surgical intervention was performed immediately. Successful excision was completed confirming a 280 × 35 mm mass without any complications, consistent with hemangioma on histopathology. Postoperative recovery showed no recurrence or complications.

**Conclusions:**

Cardiac hemangiomas are rare and its clinical course can be varied from patient to patient. We present excision of a large cardiac mass with a high mortality risk due to its size and the patient’s spiritual beliefs.

## Background

The first cardiac hemangioma case report was published by Uskoff in 1893 [1]. Although cardiac neoplasia are rare (0.28%), most are diagnosed in autopsies, with hemangiomas being only 5% of these cases [2]. Histologically, hemangiomas are classified in the principal categories: cavernous, capillary, and arteriovenous [3]. The physiopathology varies from patient to patient, some being completely asymptomatic while others debuting with sudden death. Miao and colleagues published a revision of 67 cases of cardiac hemangiomas with an average size of 52.3 mm, the largest being 130 mm, which to this day is the largest reported. We present the following case report of a successful 280 mm × 35 mm hemangioma excision that produced a right ventricular diastolic prolapse, proving a high mortality risk of the patient.

## Case presentation

**Fig. 1:**
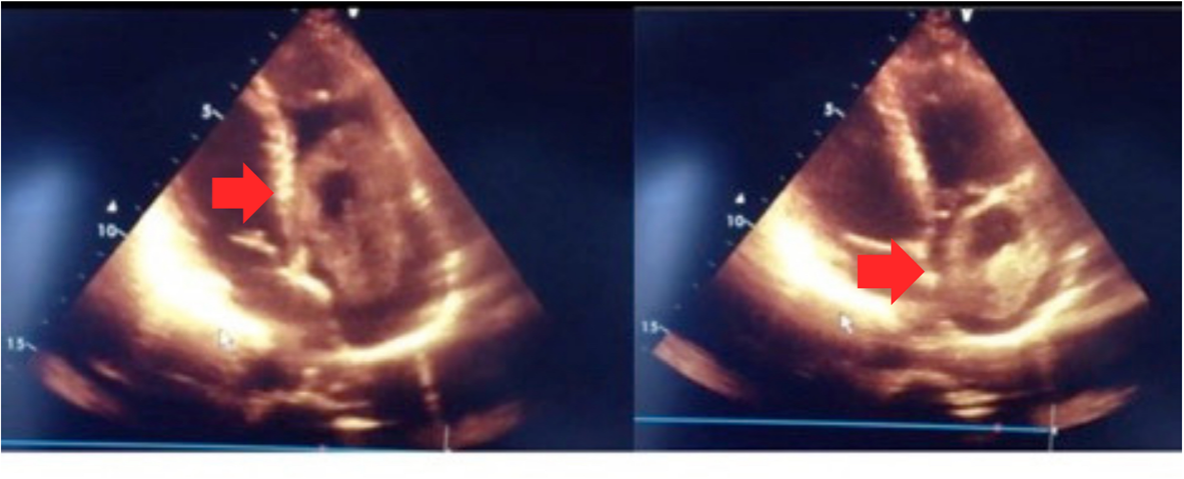
Transthoracic echocardiogram. Longitudinal view of the transthoracic echocardiogram showing a diastolic prolapse into the right ventricle (red arrows)

A 48-year-old female patient, with spiritual beliefs of Jehovah’s Witness, arrives into the emergency department due to multiple syncopal episodes the months prior, without any neurological deficits. A transthoracic echocardiogram was performed showing dilatation of the inferior vena cava and a < 50% collapse of an echogenic and heterogeneous image arising 13 cm from the right atrial opening occupying large part of it and producing a diastolic right ventricular prolapse (Fig. 1). This was a mobile longitudinal mass extending into the right chambers, despite this the left ventricular ejection fraction was preserved at 55%. Given the patient’s high complications risk due to embolism or sudden death, a surgical intervention was performed immediately.

**Fig. 2:**
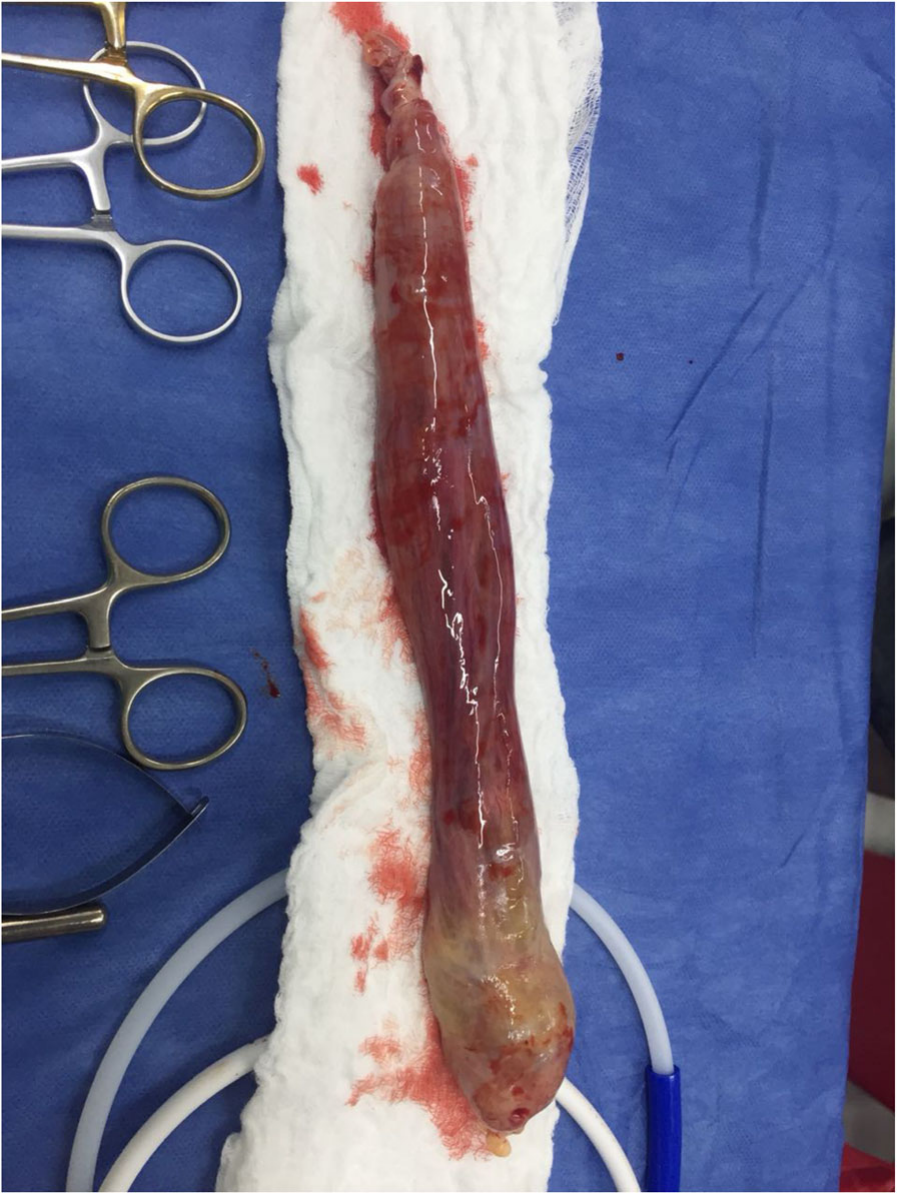
Intraoperative hemangioma. Intraoperative specimen removed, 28 cm × 3.5 cm (length x width) mass

The surgery was performed with an on-pump beating-heart cardiopulmonary bypass (CPB) without aortic cross-clamping or cardioplegic arrest. A direct superior intravenous access into the superior vena cava was achieved as well as a peripheral femoral vein access with multipurpose of active drainage #25. The right atrium was accessed via a longitudinal incision parallel to the atria-ventricular groove. The cavity was explored confirming the 280 × 35 mm (length x width) mass that extended from the inferior vena cava to the right atrium. Excision was successfully performed without any complications (Fig. 2), the cavity was then explored with no evidence of thrombus or additional masses.

The patient had a successful postoperative recovery, a postoperative transthoracic echocardiogram was performed without evidence of any recidivating masses. The patient endured a 5-day in-hospital care and was discharged without any complications. The histologic report confirmed a hemangioma of arteriovenous type. Patient follow-up 3, 6, and 12-months later reported a SF-36 of 95% (minimal disability).

## Discussion

Cardiac hemangiomas are rare primary cardiac tumors, with an incidence of only 1–2% of all cardiac primary tumors. These tumors are classified based on their histologic appearance in three categories: cavernous, capillary, and arteriovenous. The cavernous type has small vessels in its border with multiple dilations, the capillary type has numerous small-vessels similar to capillaries, while the arteriovenous has dysplasia of the arteries and veins [3]. This histological difference arises from hyperplasia or abnormal dilation of the small arterioles, venules, and capillaries [4]. They can appear at any stage of life and cardiac chamber, however they are most commonly found in the atrium. Although the clinical manifestations are varied, depending on the age of the patient, chamber localization, size, and rate of growth, most symptomatic patients have hemodynamic repercussions from valve, coronary vessels, or electrical pathways changes. Complex cases can involve other organs due to extrinsic compression, such as esophageal, tracheal, or ventricular prolapse like in this case report, resulting in high embolization risk.

Complementary images are of diagnostic use only, given that the definite diagnosis is histopathologic. However, an echocardiogram, computed tomography (CT) scan, CT angiography, or cardiac magnetic resonance image (MRI) will provide precise details that can be used in the operative approach. Given our patient’s clinical presentation and the high embolic or sudden death mortality risk, an immediate surgical excision was required. As of 2018, reports of Jehovah’s Witness supporters are estimated to be approximately 8 million worldwide, which as is common knowledge can be difficult to treat due to their beliefs in cardiac interventions [5]. However, in this case report a successful excision was completed without cardiac arrest with an on-pump beating-heart to minimize risks, achieving an excellent result with no reported complications short or long-term.

Previous reports of right chamber hemangiomas have recently been reported, however most have been of significant small sizes. Jiang et al. reported an excision of a right ventricular 68 × 50 × 26 mm mass without any hemodynamic repercussion [6]. Additionally Iida et al. also reported a 28.2 × 22.8 mass located in the right ventricle surface [7], as did Samanidis et al. with a 51 × 52 × 88 mm mass located in the right atrium without extension to any other chambers [8]. Lastly, Takahashi et al. did report a larger than usual hemangioma (60 × 60 mm) located in the left atrial appendage [9]. The importance of this case report reveals the compromise compared to other large hemangiomas, given that in our case report the mass affected the right chambers of the heart due its large size and subsequently proved a high-risk situation for the patient.

## Conclusions

The incidence of cardiac hemangioma is low and its clinical course can be varied from patient to patient. We have described a unique case of a Jehovah’s Witness patient as well as a challenging and difficult surgical case. The key to proper management is a multidisciplinary approach as well as expertise and proper communication between physicians.

## Data Availability

The dataset supporting the conclusions of this article is included within the article.

## Abbreviations

CPB: Cardiopulmonary bypass
CT: Computed tomography
MRI: Magnetic resonance image
